# Diagnosis of bladder cancer and prediction of survival by urinary metabolomics

**DOI:** 10.18632/oncotarget.1744

**Published:** 2014-02-17

**Authors:** Xing Jin, Seok Joong Yun, Pildu Jeong, Isaac Yi Kim, Wun-Jae Kim, Sunghyouk Park

**Affiliations:** ^1^ College of Pharmacy, Natural Product Research Institute, Seoul National University, Sillim-dong, Gwanak-gu, Seoul, 151-724, Korea; ^2^ Department of Urology, College of Medicine and Institute for Tumor Research, Chungbuk National University, 52 Naesudong-ro, Heungdeok-gu, Cheongju, Chungbuk, 361-711, Korea; ^3^ Section of Urologic Oncology, The Cancer Institute of New Jersey, Robert Wood Johnson Medical School, New Brunswick, New Jersey, USA.

**Keywords:** bladder cancer, metabolomics, LC-MS, diagnosis, multivariate analysis

## Abstract

Bladder cancer (BC) is a common cancer but diagnostic modalities, such as cystoscopy and urinary cytology, have limitations. Here, high-performance liquid chromatography-quadrupole time-of-flight mass spectrometry (HPLC-QTOFMS) was used to profile urine metabolites of 138 patients with BC and 121 control subjects (69 healthy people and 52 patients with hematuria due to non-malignant diseases). Multivariate statistical analysis revealed that the cancer group could be clearly distinguished from the control groups on the basis of their metabolomic profiles, even when the hematuric control group was included. Patients with muscle-invasive BC could also be distinguished from patients with non-muscle-invasive BC on the basis of their metabolomic profiles. Successive analyses identified 12 differential metabolites that contributed to the distinction between the BC and control groups, and many of them turned out to be involved in glycolysis and betaoxidation. The association of these metabolites with cancer was corroborated by microarray results showing that carnitine transferase and pyruvate dehydrogenase complex expressions are significantly altered in cancer groups. In terms of clinical applicability, the differentiation model diagnosed BC with a sensitivity and specificity of 91.3% and 92.5%, respectively, and comparable results were obtained by receiver operating characteristic analysis (AUC = 0.937). Multivariate regression also suggested that the metabolomic profile correlates with cancer-specific survival time. The excellent performance and simplicity of this metabolomics-based approach suggests that it has the potential to augment or even replace the current modalities for BC diagnosis.

## INTRODUCTION

Bladder cancer (BC) is the seventh most common cancer worldwide in men and the 17th most common cancer in women [1]. At the time of diagnosis, about 70–80% of BCs are non-muscle-invasive bladder cancers (NMIBCs), while the remaining 20–30% is muscle-invasive bladder cancers (MIBCs). Although these BC types both originate from the urothelium in the urinary bladder, they have distinct clinical characteristics. Non-muscle-invasive bladder cancer (NMIBC) is associated with good survival compared to other malignancies, but 30-50% of patients with NMIBC will eventually experience recurrence after transurethral resection (TUR) of the primary tumor, and 10–20% will progress to muscle-invasive bladder cancer (MIBC) [2]. In the case of MIBC, while radical cystectomy, radiation therapy, and chemotherapy are considered to be effective therapies, patients with MIBC often have poor outcomes despite systemic treatment [3]. Therefore, to manage BC properly, it is essential to obtain a precise and early diagnosis of BC.

Diagnostic strategies for BC have historically relied on the combination of cystoscopy and urinary cytology. However, the cystoscopic procedure is costly, invasive, and uncomfortable. While urinary cytology is a convenient method for diagnosing BC, its sensitivity is low, which reduces its reliability. Therefore, new and convenient diagnostic approaches that can distinguish BC from non-malignant conditions and MIBC from NMIBC are needed.

Metabolomics is a relatively new scientific field for studying the biochemical processes that involve metabolites. As metabolites are present in readily-available biofluids, metabolomics has been applied to the diagnosis of many cancers, such as ovarian cancer [4], pancreatic cancer [5, 6], and leukemia [7]. Recent application also includes direct profiling of tissue metabolites without any extraction step, using high-resolution magic angle spinning NMR method [8, 9]. Efforts have been also made to apply metabolomics to tissue imaging based on local metabolite patterns [10, 11]. The clinical applications of metabolomics are expected to grow further, considering that the vast majority of clinical diagnostic methods are based on small molecules [12].

Of the various cancers, BC seems to be ideal for urinary metabolomics-based diagnosis, as urine can directly contact the cancer lesion in the bladder. Moreover, urine collection can be made conveniently and its metabolomics study is non-invasive. For these reasons, there have been some metabolomics applications to BC diagnosis with various platforms, and LC-MS, GC-MS, and NMR metabolomics have been used to suggest that metabolomic approach has a potential for early or accurate diagnosis of BC [13-16]. However, most of these studies used small sample sizes and did not properly consider possible confounding effects of benign hematuria. In addition, these studies did not confirm the metabolic markers based on other experimental approach. Furthermore, the ability of metabolomic profiles to predict the survival of patients with BC has not yet been assessed.

In the present study, a metabolomics approach using high-performance liquid chromatography-mass spectrometry (HPLC-MS) was carried out. Of all metabolomics-based diagnosis studies in BC, this study employed the largest number of patients to date (138 cancer patients and 121 controls). In addition, a substantial proportion of the control group consisted of patients with benign hematuria (n = 52). Rigorous statistical cross-validation and comparisons with microarray gene expression data revealed that the metabolomics-based diagnostic approach that was developed performed well, and that the distinguishing markers relate to glycolysis and fatty acid betaoxidation. Metabolomics could also be used to distinguish between NMIBC and MIBC and predict the survival of patients with BC.

## RESULTS

### Baseline characteristics

Supplementary Table S1 lists the baseline characteristics of enrolled patients and controls. The patients were 65.64 ± 12.65 years old on average and consisted of 112 males and 26 females. The controls were 64.31 ± 9.18 years old on average and consisted of 77 males and 44 females. Thirty-one, sixty-three, and forty-four patients had grade G1, G2 and G3 cancer, respectively and 48, 35, 25, 10 and 20 patients had stage Ta, T1, T2, T3, and T4 cancer, respectively. Seventeen (12.3%) patients had one or more lymph-node metastases and nine (6.5%) had distant metastasis. The median follow-up period was 37.1 months. Of the 138 patients with BC, 83 (60.1%) had NMIBC and 55 (39.9%) had MIBC.

### Differentiation between BC patients and controls, and between NMIBC and MIBC based on urine metabolomic profile

**Figure 1:**
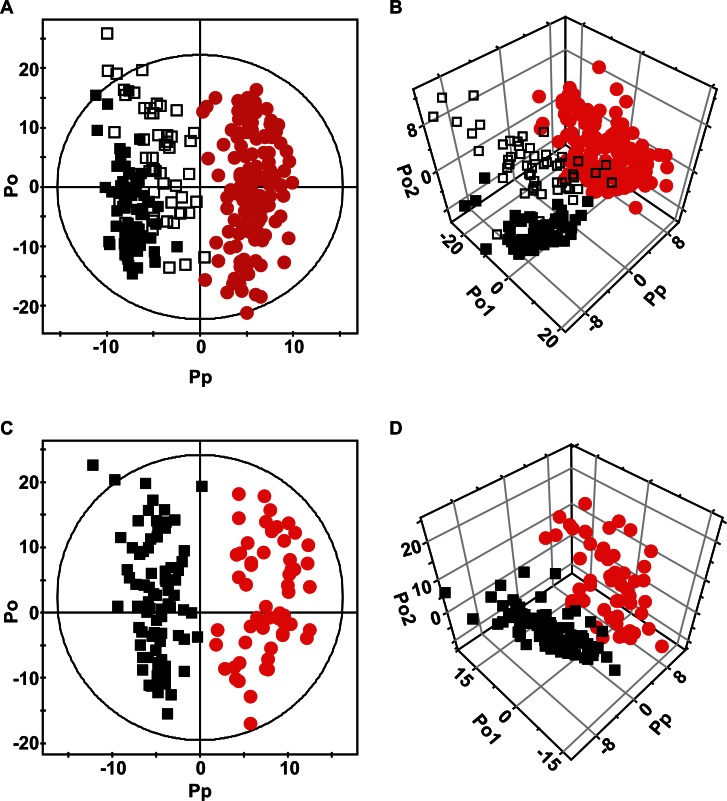
OPLS-DA score plots (A & C) and 3D scatter plots (B & D) Each symbol represents the metabolomic profile of an individual sample. (A & B) Comparison of normal controls (black) and patients with bladder cancer (red). The open black boxes represent the control patients with benign hematuria. (C & D) Comparison of the two types of bladder cancer. NMIBCs are represented by black boxes while MIBCs are represented by red dots.

Representative HPLC-MS chromatograms of the urine samples of the healthy control subjects and the patients with NMIBC or MIBC are shown in Supplementary Figure S1. The peaks were very well resolved and were evenly dispersed across the entire retention time domain, showing high qualities of the raw data. In addition, the overall peak profiles of the three groups looked quite different, which suggested that these profiles could be used to discriminate between the groups. For the holistic treatment of these data, multivariate analysis was used to identify the metabolomic differences between the groups. An orthogonal projections to latent structure-discriminant analysis (OPLS-DA) model was obtained with one predictive and two orthogonal components, and it gave good separation between the normal subjects and the cancer patient group (NMIBC and MIBC were combined) (Figure 1A-B) (R^2^ = 0.878 and Q^2^ = 0.662). Notably, 43% of the normal group were patients with hematuria (n = 52) who did not have cancer (the open black boxes in Figure 1A-B). These patients are an important control because hematuria is frequently found among BC patients, and thus, its presence in normal patients could be a possible confounding factor in the cancer diagnosis. The results showed that the urinary metabolomics approach can differentiate the normal hematuric patients from patients with cancer. We also performed the differentiation between the two subgroups of cancer, NMIBC and MIBC (Figure 1C-D), and the orthogonal projections to latent structure-discriminant analysis (OPLS-DA) model with one predictive and two orthogonal components separated the cancer groups reasonably well (R^2^ = 0.875 and Q^2^ = 0.355). These findings indicate that LC-MS analysis of urine can help the diagnosis of BC and differentiation between its subtypes.

### Identification of cancer-specific metabolites

**Figure 2:**
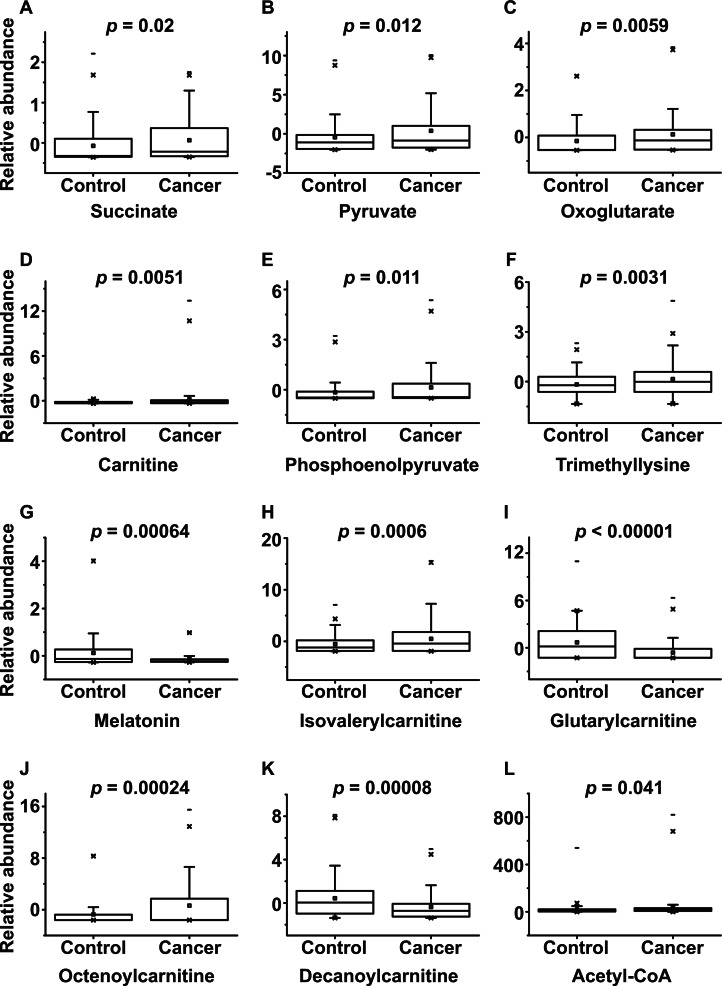
Box plots of the levels of potential metabolomic markers that could be used to distinguish BC patients from control subjects The *p*-values of Student's *t*-test are indicated.

The successful distinction of normal and cancer groups led us to search for the specific metabolites that contributed to the metabolomic differences between these groups. Based on the OPLS-DA model described above, the signals that had high correlation and signal-to-noise ratio values were selected. These included the signals at 119.04, 130.05, 147.03, 162.11, 169.01, 189.16, 233.11, 246.17, 276.14, 286.20, 316.25, and 810.13 m/z (Table 1). The molecules responsible for these signals were identified by analyzing the MS/MS spectra and comparing them to the spectra of known standard compounds (Table 1). Several of them belonged to carnitine and acylcarnitines (carnitine, isovalerylcarnitine, glutarylcarnitine, octenoylcarnitine, and decanoylcarnitine), while others related to the last steps of glycolysis (phosphoenolpyruvate and pyruvate) or the TCA cycle (acetyl-CoA, succinate, and oxoglutarate). To confirm that the control and cancer groups differed in terms of the urinary levels of these metabolites, Student's *t*-tests were performed (Figure 2). Indeed, the two groups differed significantly in terms of all of these markers.

**Table 1:** Potential urine biomarkers of bladder cancer (1-12) and mRNA expression of related genes in BCs relative to control tissue (13-15)^a^.

NO.	m/z	Adduct	Metabolite/gene	Element composition/symbol	*p*-value^b^	Trend^c^
1	119.0362	[M+H]^+^	Succinate	C_4_H_6_O_4_	2.0E-02	↑
2	130.0491	[M+ACN+H]^+^	Pyruvate^d^	C_3_H_4_O_3_	1.2E-02	↑
3	147.0285	[M+H]^+^	Oxoglutarate^d^	C_5_H_6_O_5_	5.9E-03	↑
4	162.1109	[M+H]^+^	Carnitine	C_7_H_15_NO_3_	5.1E-03	↑
5	169.0083	[M+H]^+^	Phosphoenolpyruvate	C_3_H_5_O_6_P	1.1E-02	↑
6	189.1602	[M+H]^+^	Trimethyllysine	C_9_H_20_N_2_O_2_	3.1E-03	↑
7	233.1104	[M+H]^+^	Melatonin	C_13_H_16_N_2_O_2_	6.7E-04	↓
8	246.1695	[M+H]^+^	Isovalerylcarnitine	C_12_H_23_NO_4_	6.0E-04	↑
9	276.1441	[M+H]^+^	Glutarylcarnitine	C_12_H_21_NO_6_	2.2E-09	↓
10	286.2010	[M+H]^+^	Octenoylcarnitine	C_15_H_27_NO_4_	2.4E-04	↑
11	316.2465	[M+H]^+^	Decanoylcarnitine	C_17_H_33_NO_4_	7.9E-05	↓
12	810.1328	[M+H]^+^	Acetyl-CoA	C_23_H_38_N_7_O_17_P_3_S	4.1E-02	↑
13	-		Carnitine palmitoyltransferase	CPT1A	8.4E-03	↑
14	-		Carnitine acylcarnitine translocase-like protein (CACL)	SLC25A29	3.9E-03	↑
15	-		Dihydrolipoyl dehydrogenase (Pyruvate dehydrogenase complex)	DLD	1.0E-07	↓

^a^The mRNA expression levels are from our microarray experiment, which was reported previously 23.

" id="tabb">^b^ The *p*-values are from Student's t-tests.

" id="tabc">^c^ The trend of the marker levels in the BC group. ↑ and ↓ indicate increased and decreased levels, respectively in the cancer group.

" id="tabd">^d^ Tentative identification.

### Diagnostic performance of the metabolomics-based model

**Figure 3:**
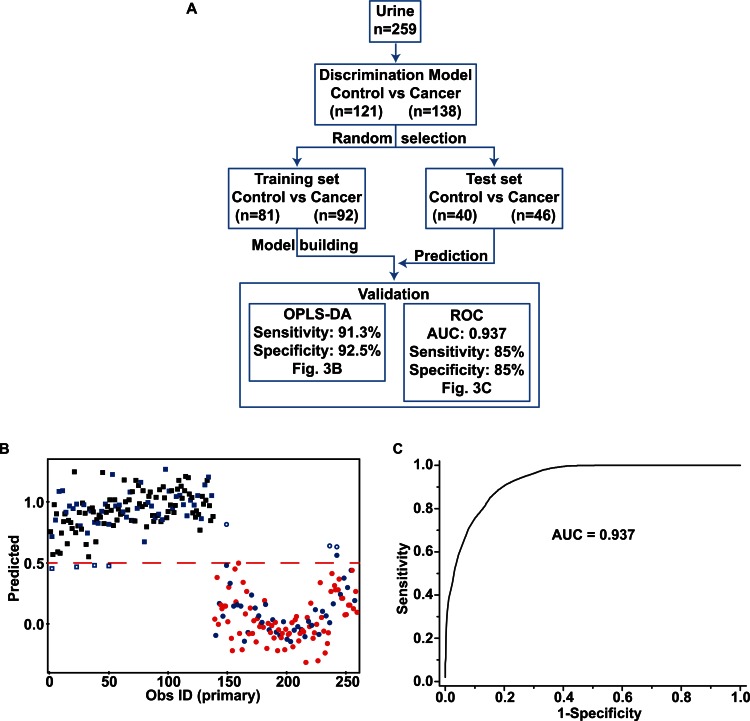
Cross-validation with an OPLS-DA model and multivariate ROC analysis (A) Schematic depiction of the overall procedure of cross-validation analysis. The test set was created by randomly selecting one third of the entire sample. A prediction model was built with the rest of the samples (training set), after which the models were used to predict the cancer status of the test set. Diagnostic performance was assessed by either OPLS-DA or PLS-DA based ROC curve analysis. (B) Prediction of the cancer status using the OPLS-DA model. The boxes represent BC patients while the dots represent control subjects. The green samples represent the test set. The samples represented by open green symbols are mispredicted samples. The dichotomic decision of prediction was made by using the *a priori* value of 0.5 for the Y variable from the OPLS-DA model. Of the 46 cancer samples, 42 were predicted to be from cancer patients (91.3% sensitivity) while 37 of the 40 control samples were predicted to be from control subjects (92.5% specificity). (C) PLS-DA-based ROC curve analysis using the same test set revealed a sensitivity of 85% and specificity of 85%. The area under the curve (AUC) value was 0.937.

To evaluate the performance of the multivariate model in the diagnosis of BC, cross-validation was carried out. As many as one third of the total samples (40 control subjects and 46 cancer patients) were randomly picked and used as the test set, and the prediction model was built from the rest of the samples (training set) (Figure 3A). The models were then used to predict the diagnoses of the test set subjects, and the comparison with the actual diagnoses yielded sensitivity and specificity values. This cross-validation is important for determining the practical applicability of metabolomics-based diagnostic approaches; however, this has not always been performed in other BC metabolomics studies. The OPLS-DA model predicted that 42 of the 46 cancer samples were from cancer patients, while 37 of the 40 normal samples were predicted to be from normal subjects. Thus, the prediction model had a sensitivity of 91.3% and a specificity of 92.5% (Figure 3B). A partial least square-discriminant analysis (PLS-DA)-based multivariate receiver operating characteristic (ROC) curve was also generated with the same training and test sets used with the OPLS-DA prediction test (Figure 3A), because receiver operating characteristic (ROC) has been conventionally used to evaluate diagnostic performance in clinical research. The resulting area under the curve (AUC) was 0.937 and the sensitivity and specificity values were both 85% (Figure 3C). Thus, the two validation methods diagnosed cancer with comparable sensitivity and specificity (85–92.5%). The ability of the OPLS-DA model to differentiate between NMIBC and MIBC was assessed in a similar way. The OPLS-DA model predicted 24 out of 28 test NMIBC samples correctly (85.7% accuracy), and 9 out of 18 MIBC test samples correctly (50% accuracy) with 71.7% overall accuracy (Supplementary Figure S2).

### Survival prediction

**Figure 4:**
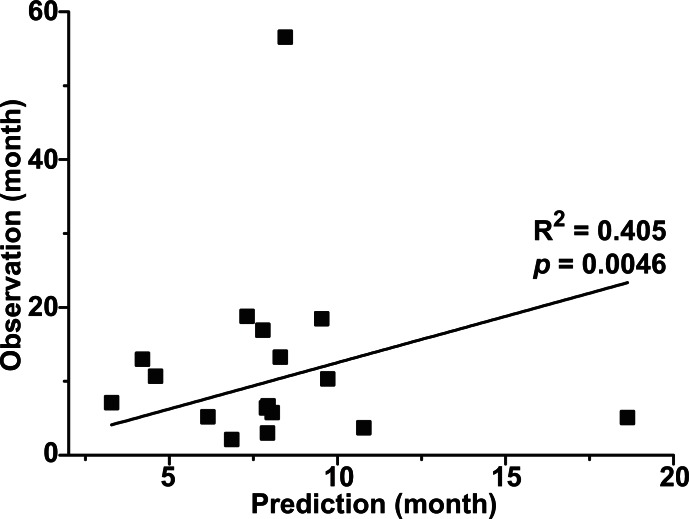
Prediction of cancer-specific survival time The PLS prediction model was obtained with three components (R^2^ = 0.991, Q^2^ = 0.404). The X-axis corresponds to the predicted values that were calculated by using the PLS regression followed by the leave-one-out prediction. The Y-axis corresponds to the actual cancer-specific survival time. The R-squared value of the linear regression is 0.405 and the *p*-value is 0.0046.

In clinical situation, the prediction of survival of cancer patients after diagnosis is critical. However, none of the previous metabolomics studies on BC have asked whether metabolomics can be used to predict survival time. This probably reflects the heterogeneity of the study populations, the fact that various treatment modalities were used, and the difficulties associated with following up enough patients for the entire duration of survival. In the present study, 34 of the 55 patients with MIBC (61.8%) underwent radical cystectomy while 26 (47.3%) received systemic chemotherapy. To date, 30 (54.5%) patients remain alive, 23 (41.8%) have died, and two (3.6%) were lost during follow-up. Of the 23 patients who died, 17 died due to cancer-related events. To determine whether our metabolomic approach could be used to predict post-diagnosis survival time, a multivariate partial least square (PLS) regression was applied for the 17 patients who died. The partial least square (PLS) prediction model was built with three components (R^2^ = 0.991, Q^2^ = 0.404) using the metabolomic profile as independent variables and the cancer-specific survival time as a dependent variable. Then, the individual cancer specific survival time was predicted using leave-one-out analysis, and the predicted values were compared with the actual survival time. The analysis gave R^2^ value of 0.405 with *p* = 0.0046 between the predicted and actual values (Figure 4), suggesting that the metabolomic profile might be useful in predicting the cancer-specific survival time of BC patients.

## DISCUSSION

BC is usually diagnosed in clinical practice by urinary cytology and cystoscopy. Although urinary cytology is convenient and detects BC with high specificity, its diagnostic ability is rather disappointing due to its low sensitivity (40–76%) [17]. Moreover, cystoscopic examination is highly invasive and relatively expensive, and thus, majority of the patients would be distressed. Therefore, new diagnostic tools that can distinguish BC from non-cancerous conditions with better sensitivity and specificity are needed. To this end, a number of urine-based tests have been developed. These include tests based on bladder tumor antigen (BTA), nuclear matrix protein 22 (NMP22), urine fibrin and fibrinogen degradation products (FDP), ImmunoCyt, and FISH (UroVysion) [18, 19]. However, the diagnostic capability of all of these tests is insufficient, and can replace cystoscopy or urinary cytology [20]. The urinary metabolomics-based diagnostic approach described in the present study may be more promising as it is clinically relevant, performs well, and is convenient. The clinical relevance of this approach is based on the fact that urine is stored in the bladder and is in direct contact with bladder tissue. Thus, its metabolomic profile may closely reflect the status of the bladder tissue, making it more clinically relevant for BC diagnosis than the blood samples used in some studies [21, 22]. With regard to its diagnostic performance, the high sensitivity and specificity shown in this study using a large number of patients (> 250) gives reliability on its performance. Especially with the high sensitivity of above 85% without compromising the specificity, it can be compared with urinary cytology which suffers from the low sensitivity. It is also important that any diagnostic test should be convenient and quick in real practice and that the diagnostic decision can be made relatively easily. Our metabolomics approach only needs 5 μL of urine (1/5000th of the volume of the typical urine sample) which can be readily obtained during a routine check-up without affecting the original tests. Moreover, compared to the cystoscopy involving local anesthesia and pain, our urinary metabolomics-based diagnostic approach is non-invasive. The sample can be analyzed in 35 minutes and the decision can be made relatively quickly and objectively, as it does not require the expertise of experienced pathologists. Given these merits, this urinary metabolomics-based diagnostic approach may have the potential to augment or even replace the cytology or cystoscopic diagnostic modalities that are currently being used.

**Figure 5:**
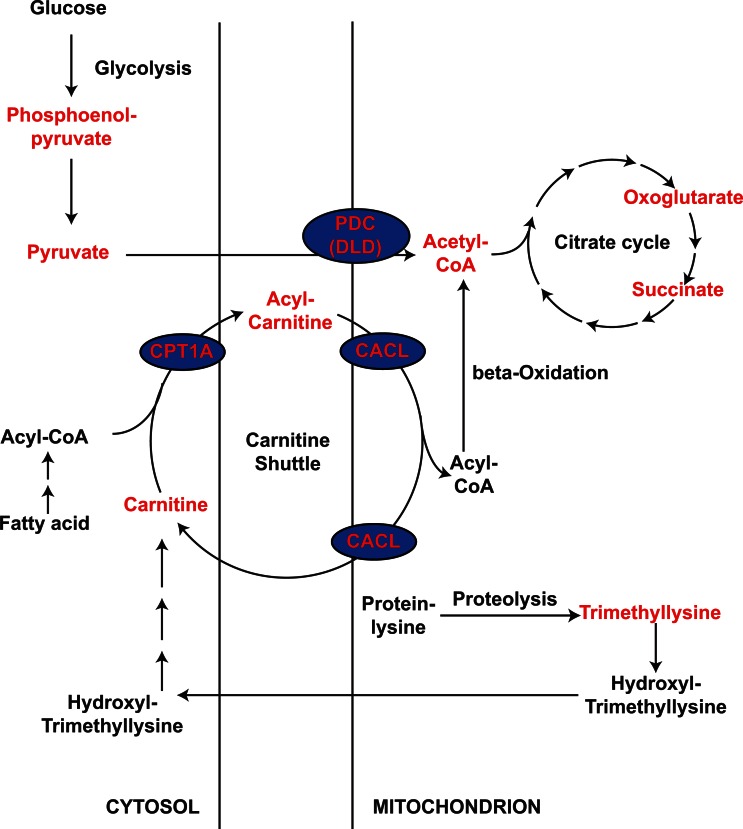
Pathways that may be altered in patients with BC compared to control subjects The metabolites detected in this study are indicated in red. The genes whose levels are modulated in our microarray analysis are indicated in blue ellipses.

The present study showed that the cancer group has elevated levels of urinary acetyl-CoA and carnitine, and several acylcarnitines were found to contribute to the differentiation between the cancer and control groups. As carnitine is essential for the entry of fatty acid into the mitochondria for oxidation, and acetyl-CoA is the final product of this oxidation event, these results suggest that fatty acid oxidation might be an important factor in determining the cancer status. We have previously published microarray analyses of BCs [23]: when we examined the gene expression levels of the enzymes involved in fatty acid oxidation, we found that BCs generally expressed significantly higher levels of the carnitine palmitoyltransferase 1A (CPT1A) than control tissues (*p* = 0.0084; Table 1). Carnitine palmitoyltransferase is a key protein that uses carnitine to transfer fatty acid into mitochondria for oxidation. In addition, the increase was more significant in MIBC (*p* = 0.0003) than NMIBC (*p* = 0.089), and its level was different significantly between the two types of cancer (*p* = 0.028) (Supplementary Figure S3A). Therefore, carnitine palmitoyltransferase 1A (CPT1A) expression may also correlate with the aggressiveness of BC, which may be an interesting subject for future studies. Supporting it is that in highly metastatic alveolar rhabdomyosarcoma cancer cells, CPT1A expression correlates with cell motility [24]. It is also interesting to see that many efforts have been made to develop inhibitors of CPT as anticancer agents [25, 26]. Our observations suggest that such inhibitors may be useful in BC treatment. Our microarray data also showed that BCs expressed carnitine acylcarnitine translocase-like protein (CACL, gene symbol: *SLC25A29*), another enzyme involved in fatty acid transport into mitochondria [27, 28], at higher levels than control tissues (*p* = 0.0039; Table 1). Both MIBCs and NMIBCs expressed significantly higher levels of carnitine acylcarnitine translocase-like protein (CACL) than control tissues (*p* = 0.0016 and 0.016, respectively, Supplementary Figure S3B), but the expression levels in MIBC and NMIBC was not different (*p* = 0.647). Although not so much study has been done for CACL as CPT1A, strategies that target it may also have therapeutic potential in both subtypes of BC. Thus, along with other studies implicating fatty acid oxidation in various carcinogeneses [29-31], our metabolomics study and microarray analysis indicate that betaoxidation may play an important role in BC tumorigenesis and possibly aggressiveness. The level of acetyl-CoA, another molecule in betaoxidation, can be affected by input from pyruvate *via* the pyruvate dehydrogenase complex (PDC). We therefore examined the expression of components of the pyruvate dehydrogenase complex (PDC), and found that the third component of the complex, dihydrolipoyl dehydrogenase (DLD), is significantly reduced in bladder cancer (*p* < 10^-7^; Table 1). This suggests that the higher acetyl-CoA levels in BC are largely due to elevated betaoxidation, rather than the result of conversion from pyruvate. This suggestion is consistent with the Warburg effect in most cancer cells [32], where pyruvate is converted to lactate rather than acetyl-CoA. The overall pathways affected by BC are summarized in Figure 5.

Several other studies have examined the metabolomics profiles in BC but most involved fewer than 45 cancer patients [13-16, 21, 22], except the one with 83 cancer patients and 51 controls [33]. In addition, the lack of cross validation involving a test set in many of the studies may have limited the application of the study in real clinical situation. By comparison, the present study has by far the largest number of patients in both the cancer (n = 138) and control (n = 121) groups, which suggest that the statistical results are more reliable. In addition, we performed OPLS-DA cross-validation analysis using as much as one third of the entire sample set as the test set. Indeed, our test set (n = 40 for the control group and n = 46 for the cancer group) was larger than or comparable to the groups used in most of the studies described above. In relation to this, one study did objectively evaluate the predictive value of their analysis platform with an independent test set [22]. They found that their OPLS-DA model had 100% sensitivity and specificity. However, only 20 patients comprised the entire BC group in this study. This suggests that these performance data should be interpreted with care. Considering our and other metabolomics reports [13-15, 34], more realistic sensitivity and specificity estimates would probably be about 85–92.5%. Equally importantly, patients with benign hematuria were included in the control group in the present study to ensure that our model would continue to differentiate between BC patients and controls in the presence of hematuria. Since patients with BC usually present with hematuria, but hematuria can also be present in patients without cancer, hematuria can be a serious confounding variable. Indeed, the control patients with hematuria clearly clustered between the cancer patients and the controls without hematuria (see Figure 1A-B). Despite the presence of many patients with benign hematuria, our model could differentiate between the control and cancer groups with excellent specificity and sensitivity. Previous studies with hematuric patients either did not include them in the control group [21] or analyzed serum, which may not be as confounded by hematuria as urine-based analyses [22]. Thus, the inclusion of patients with benign hematuria in the present study suggests that the model we developed is likely to be reliable in a real clinical situation.

## MATERIALS AND METHODS

### Chemicals

Acetonitrile was purchased from Honeywell Burdick & Jackson (Morristown, NJ) and formic acid was obtained from Fluka (St. Louis, MO). All other solvents were of HPLC grade.

### Patients and urine samples

A total of 259 subjects, 138 with primary urothelial carcinoma of the urinary bladder and 121 controls, were enrolled in the study. Controls consisted of 69 healthy people who visited the hospital for medical check-ups and 52 patients with microscopic hematuria that was due to non-malignant conditions. The controls were selected so that their ages were similar to the ages of the patients with cancer. The collection and analysis of all samples were approved by the Institutional Review Board of Chungbuk National University and written informed consent was obtained from each subject (IRB approval number 2006-01-001). Urine samples were collected in the morning and centrifuged at 25,000 rpm for 15 min. The supernatant and sediment were aliquoted separately into Eppendorf tubes and stored at -20 °C until use. All primary tumor samples were obtained from patients who underwent transurethral resection (TUR) or radical cystectomy. All were histologically verified to have urothelial carcinoma. Tumors were staged and graded according to the 2002 TNM classification and the 1973 WHO grading system, respectively. The biospecimens for this study were provided by the Chungbuk National University Hospital, a member of the National Biobank of Korea, which is supported by the Ministry of Health, Welfare and Family Affairs.

### Liquid chromatography-mass spectrometry (LC-MS)

The frozen urine samples were thawed and then centrifuged at 15,000 × g for 10 min at 4 °C to remove particulate matter. Chromatographic separation was performed on a Kinetex C18 column (2.6 µm, 100 × 4.6 mm; Phenomenex, USA) by using an Agilent 1200 Infinity Series liquid chromatography system. The column temperature was 35 °C with a flow rate of 0.35 mL/min and the autosampler cooler temperature was set at 4 °C with an injection volume of 5 µL. Analytes were eluted with a mobile phase composed of 0.1% formic acid in water (A) and acetonitrile with 0.1% formic acid (B). Gradient conditions were as follows: 0–14 min gradient 5–25% B, 14–19 min gradient 25–40% B, and 19–23 min gradient 40–95% B. After this, the solvent composition was maintained at 95% B for 6 min, followed by a return to the starting conditions and re-equilibration of the column for 6 min. Mass spectrometry experiments were performed on a Q-TOF (6530 Accurate-Mass, Agilent Technologies, Santa Clara, CA) equipped with ESI sources. Data were acquired in positive mode. The measurement conditions were as follows: ESI source voltage of 4 kV, gas temperature of 350 °C, sheath gas flow of 12 L/min, nebulizer gas at 30 psi. The scan range was 85-1000 *m/z*.

### Data processing and statistical analysis

The LC-MS raw data were converted and processed by using MZmine 2.10 (mzmine.sourceforge.net) as described in a previous report [35]. Briefly, chromatograms were built and peaks were recognized by using the local minimum search function, and the ion intensities, matching *m/z,* and retention time were grouped into peak lists. Later, these peak lists were exported individually and imported into MetaboAnalyst (www.metaboanalyst.ca). The peaks were aligned and normalized by the sum of all detected peaks. The processed and normalized data were imported into SIMCA-P (Umetrics, Umea, Sweden) for multivariate statistical analysis. To distinguish between all of the groups (control, NMIBC, and MIBC), PLS-DA was performed. OPLS-DA was used for one-to-one distinction between any two groups and marker detection [36, 37]. The survival prediction was performed by using PLS regression using the metabolite profile as the independent variable. The sensitivity and specificity of the method used to distinguish between controls and cancer patients were calculated by first removing one third of the samples, which served as a test set. The multivariate model obtained with the remaining samples was then used to predict the cancer status of the samples. All of the multivariate models were constructed by iterative procedures until the predictability value (Q^2^) stopped increasing.

## SUPPLEMENTARY FIGURES AND TABLES


