# Supplementary material for: Diagnosis of bladder cancer and prediction of survival by urinary metabolomics
Source: Oncotarget. 2014 Feb 17;5(6):1635–45. doi: 10.18632/oncotarget.1744 (PMC4039236; doi:10.18632/oncotarget.1744)
Supplement: Supplementary file 1 [file oncotarget-05-1635-s001.pdf]

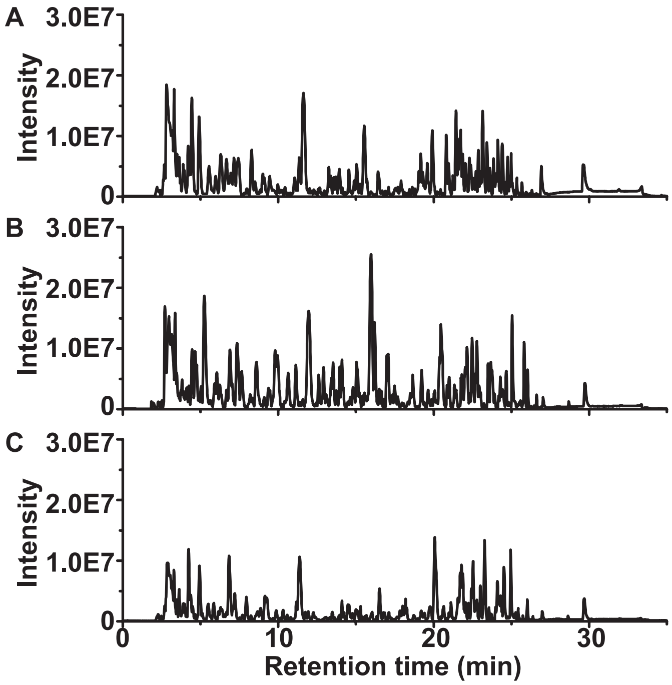

Supplementary Figure 1: Representative HPLC-QTOF/MS base peak chromatograms of human urine samples. (A) A healthy individual. (B) A patient with NMIBC. (C) A patient with MIBC.

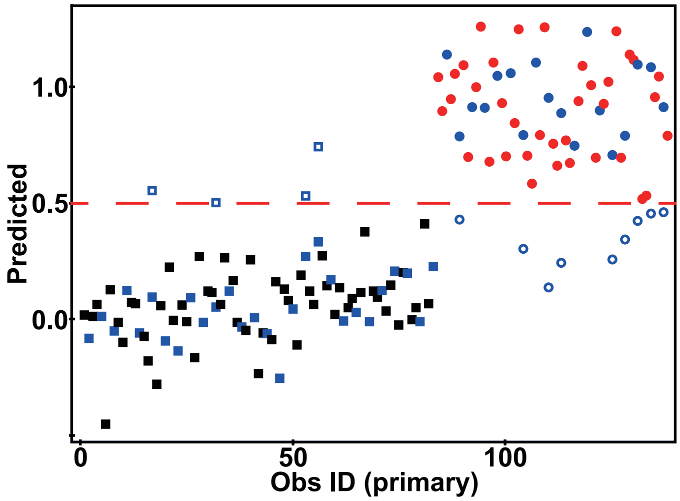

Supplementary Figure 2: Cross-validation of the differentiation between NMIBC and MIBC. The test set was created by randomly selecting one third of the each group. A prediction model was built by using the rest of the samples (training set), after which the NMIBC/MIBC status of the test set was predicted. The diagnostic performance was assessed by using the OPLS-DA model. Boxes represent NMIBC patients while the dots represent MIBC patients. The samples in blue represent the test set. The samples in open blue symbols are mispredicted samples. The dichotomic decision of prediction was made by using the *a priori* value of 0.5 for the Y variable from the OPLS-DA model.

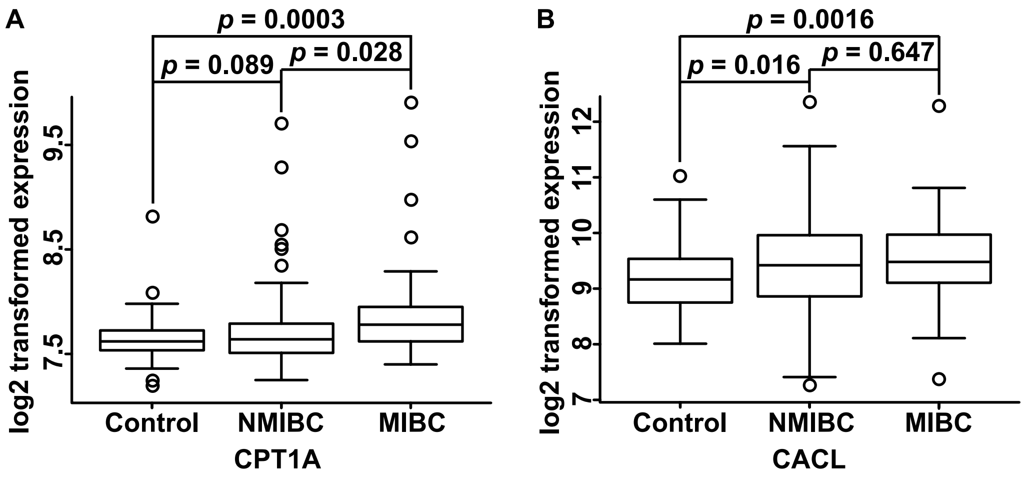

Supplementary Figure 3: Box plots of the related mRNA levels. The *p*-values of Student's *t*-tests are indicated.

Supplementary Table 1. Baseline clinical and pathological features of the patients with bladder cancer.

Variables (%)	BC cases	Controls	Healthy controls	Benign hematuria controls
Number	138	121	69	52
Mean age ± SD	65.64 ± 12.65	64.31 ± 9.18	64.28 ± 7.82	64.37 ± 10.81
Gender				
Male	112 (81.2)	77 (63.6)	41 (59.4)	36 (69.2)
Female	26 (18.8)	44 (36.4)	28 (40.6)	16 (30.8)
Grade				
G1	31 (22.5)			
G2	63 (45.7)			
G3	44 (31.9)			
T stage				
Ta	48 (34.8)			
T1	35 (25.4)			
T2	25 (18.1)			
T3	10 (7.2)			
T4	20 (14.5)			
N stage				
N0	121 (87.7)			
N(1–3)	17 (12.3)			
M stage				
M0	129 (93.5)			
M1	9 (6.5)			
Median follow-up period (months)	37.1 ± 17.5			
